# State-of-the-art surgery for ulcerative colitis

**DOI:** 10.1007/s00423-021-02295-6

**Published:** 2021-08-28

**Authors:** Shanglei Liu, Samuel Eisenstein

**Affiliations:** Department of Surgery, UC San Diego Health System, 3855 Health Sciences Dr. #0987, La Jolla, CA 92093 USA

**Keywords:** Ulcerative colitis, Surgery, Colectomy, Ileal pouch, IPAA, Continent ileostomy

## Abstract

Ulcerative colitis (UC) is an autoimmune-mediated colitis which can present in varying degrees of severity and increases the individual’s risk of developing colon cancer. While first-line treatment for UC is medical management, surgical treatment may be necessary in up to 25–30% of patients. With an increasing armamentarium of biologic therapies, patients are presenting for surgery much later in their course, and careful understanding of the complex interplay of the disease, its management, and the patient’s overall health is necessary when considering he appropriate way in which to address their disease surgically. Surgery is generally a total proctocolectomy either with pelvic pouch reconstruction or permanent ileostomy; however, this may need to be spread across multiple procedures given the complexity of the surgery weighed against the overall state of the patient’s health. Minimally invasive surgery, employing either laparoscopic, robotic, or transanal laparoscopic approaches, is currently the preferred approach in the elective setting. There is also some emerging evidence that appendectomy may delay the progression of UC in some individuals. Those who treat these patients surgically must also be familiar with the numerous potential pitfalls of surgical intervention and have plans in place for managing problems such as pouchitis, cuffitis, and anastomotic complications.

## Overview

Ulcerative colitis (UC) is one form of inflammatory bowel disease (IBD) that affects the mucosa and lamina propria of the colon. Although there is a large range of potential clinical presentation for patients with UC, typically they will have episodes of disease exacerbations separated by periods of remission. During the acute phase, exacerbation events vary from abdominal pain to fulminant colitis. Chronically, even the periods of remission may still be marked by indolent inflammation and altered bowel function [1–3].

While there are over 600,000 estimated cases of UC in North America, the underlying cause of UC remains elusive [4]. Many factors have been described to contribute to its development including genetics, hygiene, socioeconomic status, antibiotic usage, and microbiome. The most prominent of these theories is the hygiene hypothesis which attributes a decreased tolerance of the immune system to the lack of exposure to organisms earlier in life due to an upbringing in a more sterile environment. Population studies have shown higher rates of UC in developed countries or those of a higher socioeconomic class residing in more sanitary living conditions [5]. Genetics also plays an undeniable role in the development of UC as seen by the high concordant disease in monozygotic compared to dizygotic twins [6, 7]. Despite all this, the causative pathway of these contributing factors has not been elucidated.

Similarly, several surprising factors have been described as protective against the development and exacerbation of UC including smoking, alcohol intake, and history of appendectomy. Smoking has been associated with decreased UC flares, but supplemental nicotine has not been shown to have the same benefit [4]. Alcohol intake has been shown to mildly decrease the risk of UC in population studies [8]. Data from Europe has shown benefits in appendectomy in both protecting against UC and treating those medically refractory [9, 10]. While promising, the supporting evidence for these topics still falls short from proving a causative link [11].

The main colonic manifestation of UC begins in the rectum and continues with variable proximal extension. About half of patients (over 45–60%) will have disease limited to the rectum and sigmoid colon while less than half (14–37%) will have pancolitis [12, 13]. This inflammation can manifest itself in the form of hematochezia, diarrhea, abdominal pain, and, in a smaller subset of patients, constipation. When left uncontrolled, these symptoms can lead to anemia, weight loss, and growth retardation and can be very detrimental to the patient’s lifestyle and mental well-being. A minority of patients (20%) will develop additional extraintestinal UC. While some of these conditions (episcleritis, peripheral arthropathy, erythema nodosum) are known to regress after or benefit from surgical treatment of their colonic UC, several other manifestations (uveitis, axial arthropathy, primary sclerosing cholangitis, pyoderma gangrenosum) may persist beyond colectomy [14].

The management of UC involves medical treatment to suppress inflammation, endoscopic surveillance for dysplasia and treatment response, and surgical resection. Although a complete colectomy may “cure” colonic manifestations of UC, the decision and timing of surgery remain a complex decision. It is estimated that up to 25–30% of patients with UC will ultimately receive a surgical resection despite medical therapy [15, 16].

## Differential diagnosis

The diagnosis of UC relies on history, physical exam, endoscopic evaluation, and histology. Of these, endoscopy plays a pivotal role in the diagnosis and surveillance of UC. Although it may be difficult to make a diagnosis of UC with endoscopic evaluation alone, the presence of skip lesions or rectal sparing disease will rule it out. A complete colonoscopy is recommended. However, that may not always be possible during an acute flare due to potential risk of perforation. In these situations, a flexible sigmoidoscopy offers a safer alternative. Furthermore, endoscopic grading systems have been established for both disease identification and progress tracking, even if their validity is a subject of ongoing debate [17]. In the acute flare, endoscopic findings range from ulceration, contact bleeding, and cobble stoning to the formation of pseudopolyps. In the chronic inflammatory phase, findings range from loss of vascular pattern, mucosal atrophy, muscular hypertrophy, and loss of haustral folds to shortening of colon [18].

A variety of conditions share similar physical manifestations of UC and must be ruled out. The most important of these conditions is Crohn’s disease (CD). This is because the management of CD is vastly different from that of UC in terms of both the medication and the surgical approaches [19]. A thorough workup of UC must include a high suspicion for CD and ensure that it is ruled out from histology, medical history, and clinical exam. For UC, histologic findings from endoscopic biopsies may demonstrate goblet cell depletion, crypt of Lieberkuhn distortion, vascular congestion, crypt branching, crypt abscess, and neutrophilic infiltration. Physical evaluation should see continuous inflammation involving the rectum with the absence of fistulizing disease.

Many organisms can cause infectious colitis mimicking UC flare. These include *Campylobacter*, *Clostridium difficile*, *Entamoeba*, *Escherichia coli* subtype O157:H7, and *Shigella*. As a result, it is standard to obtain studies for bacteria, ova, and parasites when patients present with new onset diarrheal colitis. However, a positive test for infectious colitis cannot rule out UC as the patient may have co-infections. Nevertheless, a positive result may change the management to steer away from aggressive surgical resection in favor of antimicrobial therapy [20].

Other differential diagnoses that may mimic UC include ischemic colitis, microscopic colitis, collagenous colitis, irritable bowel syndrome, and celiac disease. A good history and physical will usually point to atypical symptoms, veering away from the diagnosis of UC. When in doubt, endoscopic visual evaluation of the mucosal inflammation pattern with biopsy remains the key to diagnosis.

## Indications for surgery

Although surgery offers a curative solution for UC, medical treatment remains the primary treatment strategy. The goal of such therapy is to induce disease remission and promote mucosal healing. As such, different medications with varying immunosuppressive capabilities are used depending on the severity of disease [21]. Whereas mild disease may be treated with mesalamine suppositories/enema, oral 5-aminosalicylic acid, oral sulfasalazine, thiopurines (6-mercaptopurine, azathioprine), or topical corticosteroids [22], moderate to severe disease often requires the usage of a monoclonal antibodies such as infliximab (Remicade) or adalimumab (Humira) and the addition of intermittent oral steroid therapy [23]. For severe exacerbations, the first-line therapy is usually intravenous corticosteroids followed by careful endoscopic evaluation although early initiation of infliximab and cyclosporin has also been tried with variable success [21, 24].

There are several indications for surgery in the treatment for UC. These include fulminant colitis, toxic megacolon, perforation, inability to tolerate medical therapy, nonresponse to medical therapy, dysplasia or malignancy, stunting of growth in children, or the need to improve certain extraintestinal manifestation of UC (i.e., uveitis, pyoderma, etc.). While early surgery for ulcerative colitis has been proven to be relatively safe, often surgery is not discussed until after initiation of medical therapy and sometimes not at all [25]. In general, the indications for surgery can be viewed based on urgency and are outlined in Table 1.

**Table 1:** Surgical indications in UC

Urgent	Elective
Fulminant/toxic colitis	Medically refractory disease
Toxic megacolon	Dysplasia/cancer
Perforation	Extraintestinal manifestations
Hemorrhage	Medication intolerance
Medically refractory disease	Growth retardation

## Urgent indications for surgery

Of all indications, those which require an urgent operation are fulminant colitis, toxic megacolon, uncontrolled hemorrhage, perforation, and medication failure. These indications are not mutually exclusive of each other and can present in combination. In all cases, it is important to have close communication with the patient’s gastroenterologist to weigh the risks and benefits of starting immunosuppressive agents in the acutely ill patient versus proceeding with total abdominal colectomy.

Toxic megacolon is a life-threatening condition presenting in patients with systemic toxicity and a distended (transverse colon > 6 cm or cecum > 9 cm) and thickened colon [26]. These patients can have diarrhea or constipation and are at high risk for perforation. They present with systemic inflammatory response syndrome (SIRS) and require immediate medical attention [27]. Initial treatment is aggressive fluid resuscitation and broad-spectrum antibiotic therapy as well as proper adjustment of medications to avoid anti-diarrheal drugs. Infectious diarrhea workup needs to be initiated to guide steroid therapy; however, the decision for surgery remains a clinical one. Any signs of hemodynamic instability, peritonitis, or stagnancy in clinical improvement for 48–72 h despite maximal medical therapy are indications for surgery [28].

Fulminant colitis presents similarly with SIRS and thickened colon but without the colonic distension. Fluid resuscitation, broad-spectrum antibiotics, intravenous steroids, and infectious workup should be promptly initiated. Inpatient administration of infliximab should also be considered as there are data suggesting it is superior to intravenous steroid therapy. However, indications for urgent surgery remain an independent clinical decision based on hemodynamic instability, peritonitis, or the lack of clinical improvement despite maximum medical therapy over 48–72 h [25].

Lower gastrointestinal bleeding (LGIB) is very common in patients with UC due to the inflamed and friable colonic mucosa. Life-threatening bleeding fortunately happens less than 5% of the time. These symptoms will usually subside with effective medical treatment, and ongoing bleeding is usually a sign of medical failure. However, in roughly 10% of patients, massive gastrointestinal hemorrhage is cited as the reason for total abdominal colectomy with an additional 12% of these patients having ongoing rectal hemorrhage after [29]. There is no agreed upon criteria on how much hemorrhage is required prior to offering the patient surgery, and it should be taken in consideration with the whole clinical picture.

## Elective indications for surgery

Patients with UC carry higher than average population risks for colorectal cancer (CRC) at about 8% 30 years after diagnosis. Additionally, these patients tend to have later stage CRC when compared to the general population at time of diagnosis with nearly 20% having unresectable disease [30, 31]. The finding of CRC in UC patients is considered to increase the risk for synchronous and future disease. This is because the detected CRC likely represents a field effect of chronic inflammation on the colonic mucosa. As such, total proctocolectomy with an appropriate segment-specific oncologic mesenteric resection is recommended.

For patients with chronic UC of either 8 years in duration or significant colonic involvement, it is recommended that they undergo surveillance colonoscopy every 2 years or sooner [32]. During this procedure, random biopsies are taken due to the difficulty in visually identifying dysplasia due to chronic inflammation. If this is returned with high-grade dysplasia (HGD), if there is a suspected dysplasia-associated lesion or mass (DALM), or if there are multifocal areas of dysplasia, the recommendation is proctocolectomy due to the high risk of underlying CRC [33]. The recommendation for surgery for findings of low-grade dysplasia (LGD) remains an area of ongoing research. Investigators have reported chances of unexpected HGD or cancer in patients undergoing surgery for LGD to be between 3 and 23%. Progression of disease also varies from 5-year development of HGD or cancer to be as high as 53% to those that quote an annual cancer rate of 0.8% [34–37]. This variability is likely due to differences in grading between low- and high-grade dysplasia among pathologists. Thus, it remains a discussion of risk and surveillance strategy with the patient when LGD is discovered on surveillance colonoscopy. The discussion should take into account general health, lifestyle preference, anal sphincter function, and the resources for close high-quality endoscopic follow-up as well as the possibility of complete endoscopic removal of the dysplastic mucosa [38].

Medication failures include both failures to control disease on medication and the inability to tolerate side effects of the medication. For example, infliximab has the risk of transfusion reactions, antibody development, severe infections, hepatotoxicity, etc. Additionally, for those patients responding only to systemic corticosteroids, they are subject to a long list of serious side effects of chronic corticosteroid usage [39, 40]. In these patients, an elective resection may be the preferred alternative.

Some forms of extraintestinal manifestations of UC can improve after surgical resection. Erythema nodosum, episcleritis, and peripheral arthralgia are known to improve after proctocolectomy. This most likely has to do with the decreased circulating systemic inflammatory molecules but cannot explain why conditions such as primary sclerosing cholangitis and pyoderma gangrenosum do not respond to surgery [41, 42]. Although surgery is still not the recommended treatment for isolated extraintestinal manifestation of UC, it may be useful to consider when having the discussion for elective colectomy with the patient when presenting in combination with another cause.

Finally, it is reasonable to consider colectomy with or without an ileoanal pouch creation when discussing the issue of lifestyle with the patient. Although surgery may be accompanied with more frequent postoperative bowel movements (with a pouch reconstruction) or the creation of an ileostomy [16], for patients who wish to reduce the number of flares in their lifetime or avoid prolonged biologic therapy, the possibility of a curative operation should be discussed [43].

## Materials and methods

At high-volume centers, the majority of surgery in the elective setting is currently being performed using a minimally invasive approach [44]. There are many who advocate for laparoscopic or robotic approaches to these procedures, and much has been written on the advantages of a minimally invasive approach including less intraoperative blood loss, decreased LOS, faster return of bowel function, and improved 30-day outcomes [45–47]. A recent meta-analysis comparing robotic to laparoscopic surgery in this setting was inconclusive, finding both to be safe, but that robotic procedures took longer to perform with a nonstatistical trend towards fewer complications [48]. These types of studies are significantly hindered by small sample sizes, and thus, it is hard to draw any conclusion about superiority. As to the procedural lengths, we have demonstrated that as the learning curve is overcome, the time of surgery of robotic procedures begins to approach that of laparoscopy as the advantages the robot imparts overcome some of its weaknesses [49]. As the operating theater becomes more technologically advanced and robotic platforms evolve, likely there will be some amount of robotic integration into all minimally invasive surgery in the future, ultimately making this argument a moot point.

Regardless of one’s minimally invasive approach, standard equipment includes atraumatic bowel graspers, monopolar cautery, a bipolar vessel sealing device, and staplers. While many surgeons will take large vessels with modern vessel sealers, some will instead staple with vascular-sized staples or clip and cut between. Bowel thickness should be assessed via palpation when stapling as oftentimes diseased bowel can be quite thick. We generally recommend having a flexible endoscope available in case there are concerns regarding anastomosis or rectal stump integrity.

Transanal IPAA (TaIPAA) has also been gaining in popularity. The procedures generally employ a combination of transanal and transabdominal approach to complete the proctocolectomy. Afterwards, the reconstruction can still be either a stapled or hand-sewn anastomosis with or without mucosectomy. The procedure can accurately identify the point of transection and anastomosis at the beginning of the procedure, adding to the accuracy of this technique. This approach has been shown to be successful in several studies despite being technically challenging [50, 51]. The procedure is generally performed with 2 laparoscopic teams and will employ a transanal minimally invasive surgery (TAMIS) port for the transanal dissection. There are also some who would advocate for a single-incision laparoscopic (SILS) approach through the ileostomy site or through a Pfannenstiel incision for the abdominal portion. A variety of SILS port can be employed by the surgeon in this scenario. A note of caution should be taken when adopting TaIPAA as this approach was prohibited in Norway for rectal cancers recently after unnecessarily high anastomotic leak rates and poor oncologic outcomes were described [52]. This approach was also abandoned recently by a high-volume US group after they found their functional outcomes unacceptable (data unpublished).

## Surgical techniques

Surgery in the setting of UC generally includes total proctocolectomy (TPC) with either end ileostomy (EI) or reconstruction with an ileal pouch anal anastomosis (IPAA). The standard of care IPAA is generally a J-pouch. However, there are certain situations where an S-pouch may be appropriate. The W-pouch is of historic significance although it has largely been abandoned. Some patients who are not candidates for pelvic reconstruction may also ultimately opt for a continent ileostomy. When discussing the current state of the art in this setting, the primary topics worth discussing include staging of pelvic pouch surgery, techniques of pouch creation, and technological advances in the performance of these procedures.

Appropriate staging of an IPAA is critically important in minimizing the risk of postoperative complications in the fewest possible procedures. Patients with UC are often malnourished and anemic while having been exposed to immunosuppressive medications such as steroids and biologic agents. These patients are poor candidates for the extensive amount of surgery to remove their disease and reconstruct to preserve continence. Because of this, they are high risk for infectious complications. In particular, pelvic sepsis from an anastomotic leak is known to happen in approximately 10% of cases [53]. Most importantly, patients who develop pelvic sepsis have up to a fourfold increase in pouch failure [54]. Prior to the biologic era, undiverted pouches were common. In the modern era of UC management, patients have often failed numerous biologics and have been seriously deconditioned by the time they come for surgery. As a result, the majority of IPAAs are currently created with a protective loop ileostomy. A recent analysis of high-volume IBD centers in the US showed that only 5.5% of IPAAs were undiverted [44].

Current staging strategies include 1-, 2-, 3-, and modified 2-stage approaches; this is outlined in Table 2. The 2-stage approach includes TPC with IPAA and diverting loop ileostomy (DLI) followed by ileostomy closure while the modified 2-stage approach includes subtotal colectomy (STC) with EI followed by completion proctectomy with an undiverted IPAA. The 3-stage approach includes STC with EI, followed by completion proctectomy with IPAA and DLI, finishing with an ileostomy closure. Each of these approaches has its own merits.

**Table 2:** Pouch staging

Staging	1st stage	2nd stage	3rd stage
1-stage	Total proctocolectomy with IPAA		
2-stage	Total proctocolectomy with IPAA and loop ileostomy	Ileostomy closure	
Modified 2-stage	Subtotal colectomy	Completion proctectomy with IPAA	
3-stage	Subtotal colectomy	Completion proctectomy with IPAA and loop ileostomy	Ileostomy closure

One-stage procedures are rarely done; there may be a role for this procedure in healthier patients, for example, patients undergoing their procedures for dysplasia instead of medically refractory disease. In reality, these procedures are predominantly being performed via a classical 2-stage approach [44]. Postoperative management of these patients can be complex as most will leave a rectal tube (RT) for an extended time to help minimize the back pressure of the anal sphincters on the anastomosis. Another popular strategy includes leaving a nasogastric tube (NGT) and not taking it out until the RT volume surpasses that of the NGT.

Most surgeons are still performing 3-stage procedures for patients who are ill and admitted to the hospital. This is also a common approach for patients who are on high-dose steroids and significantly deconditioned. While the 3-stage approach is more popular, some will consider the modified 2-stage approach. The clinical reasoning for this is to give the patient an opportunity to recover from the systemic effects of their disease after their STC in order to become a better surgical candidate at the time of their IPAA. Generally, surgeons who utilize this approach will delay the second stage procedure longer than that of their 3-stage procedures in an attempt the further optimize their patients for an undiverted IPAA. While there is a paucity of a high-quality prospective data comparing modified 2-stage to other approaches, a recent meta-analysis demonstrated that there was an increase in anastomotic leaks in pediatric patients undergoing a modified 2-stage approach when compared to the 3-stage approach. It also showed a lower leak rate in comparison to traditional 2-stage IPAA in patients with increasing exposure to biologics [55]. Large prospective data is still needed to truly assess the utility of this approach.

Prior to closing a DLI in an IPAA patient, surgeons will often obtain a barium or gastrografin enema, either fluoroscopically or via CT. Pouchoscopy is indicated if there is any question after radiologic evaluation and will often involve dilation of the ileal anal anastomosis which is frequently stenotic in the diverted state. Most surgeons will wait 8–12 weeks prior to considering DLI closure. Previously, it has been shown safe to close loop ileostomies within 13 days after rectal cancer surgery provided there was negative imaging for a leak [56]. The Short or Long Interval to Ileostomy Reversal After Ileal Pouch Surgery (SLIRPS) trial set out to determine if this was also feasible after IPAA. Unfortunately, this study had to be closed early due to extremely high complication rates in the early ileostomy closure arm (data unpublished). Most surgeons participating in this trial felt that these ileostomy takedowns were much more complex than rectal cancer ileostomies, likely due to the tethering of the ileum in the pelvis.

There are some situations where an ileorectal anastomosis (IRA) may be considered in lieu of an IPAA. IRA does not require the extensive pelvic dissection and is thought to lead to better bowel function, urinary function, and sexual function [57]. Short-term results are generally good, with many series showing failure rates as low as 7% at 10 years [57]. These tend to increase with time with > 50% failure rates at 20 years [58]. Caution should be taken when interpreting these data as well, since many of the larger studies are older, and patients undergoing surgery for UC have often been preselected as those who have no further medical options, and thus, the modern patient is much more likely to have failure of this approach without any hope of rescue. Some also advocate for this approach in a subset of UC patients who have primary sclerosing cholangitis (PSC) and relative rectal sparing on endoscopy. The concern for these patients is that they are at an increased risk of pouchitis and they will be better off functionally with an IRA. If these patients were to develop cirrhosis, they may also develop peristomal varices, and thus, ileostomy is not an ideal option for these patients either. These patients will require aggressive endoscopic surveillance, however, as they are at an increased risk of developing rectal cancer.

Another controversial topic is whether or not to perform an intra-anal mucosectomy during the creation of the IPAA. The reasoning behind this is to minimize the risk of inflammation of the retained rectal cuff (cuffitis). In the USA, extremely few IPAAs are performed with mucosectomy, with our own internal analysis of NSQIP-IBD data demonstrating that less than 5% of IPAAs each year will employ this technique (data unpublished). Despite this, cuffitis is still a very rare entity occurring in approximately 15% of IPAAs [59, 60]. Retrospective data has also shown that patients undergoing mucosectomy have worse functional outcomes with increases in night seepage, pad usage, and incontinence and decreases in resting and squeeze pressures for patients undergoing mucosectomy versus stapled anastomosis [61]. However, those who routinely perform mucosectomy claim better functional outcomes attributed to the complexity of the technique and their ability to better protect the anal sphincter. Often improved outcomes can be achieved by minimizing the use of surgical energy around the anal sphincter complex which can potentially prevent delayed thermal injury. Those performing IPAA must understand how to perform a mucosectomy as a necessary approach should the surgeon encounter technical difficulties during the proctectomy.

For those whom a pelvic pouch is not an option, a continent ileostomy (CI) may provide a better quality of life than an end ileostomy. Continent ileostomies include the Kock pouch (KP), Barnett continent intestinal reservoir (BCIR), and a T-pouch (TP). Continent ileostomies were extremely popular options prior to the development of pelvic pouches. But since IPAA has become the standard of care, CI creation has become surgeries that are infrequently performed. All forms of CI have a valved os which necessitates cannulation to relieve an abdominal pouch of its stool burden. Because these stomas are continent, they do not require pouching, enabling the os to be smaller and placed lower in the abdomen so that they are not as obvious. Creation of these pouches is quite complex, and generally, if one does not frequently perform these procedures, it is inadvisable to create one as there are significant risks of valve slippage, incontinence, and abdominal fistula.

There is emerging evidence that appendectomy may prevent colectomy in the setting of UC. Previously several large population studies had demonstrated a decreased risk of UC and a decreased risk of surgery in the setting of UC in patients who had previously undergone appendectomy [9–11, 62]. This information has led some to conclude that by modulating the reservoir of the colon’s microflora, one can affect the severity of their disease. There have been several small series examining the relationship between appendectomy and a reduction of disease severity including a cohort of 30 patients with proctitis [63]. In this study, 90% had a decrease in disease severity and 40% developed endoscopic disease remission at 14 months. More recently, the PASSION trial demonstrated that in 30 patients, 12 maintained clinical benefit of the appendectomy up to 12 months [10]. This cohort had more severe colitis than the previous study, which likely explains the worse overall outcome. However, they did demonstrate that patients which had appendiceal inflammation were much more likely to derive a benefit at that time point. There is little long-term data to support this practice, and it is not clear that the short-term clinical benefit is worth exposing these patients to the risk of surgery.

## Pitfalls and complications

One of the biggest challenges in these procedures is ensuring the pouch anal anastomosis reaches the pelvis without any tension. This can be particularly challenging in men, and particularly if they are obese. The increase in adipose tissue in the mesentery can increase bulk and functionally feel foreshortened. This can also be more challenging if a patient is to undergo a mucosectomy. Previously, when the majority of these procedures were performed in open fashion, it was recommended to test the reach of the bowel by bringing the most dependent portion down into the pelvis prior to transecting the rectum. This would allow one to tailor the length or the cuff so as to minimize tension on the pouch. This can be more challenging in the era of MIS surgery, but is still a good recommendation for patients where there is concern that the pouch may not reach. A good estimation of reach is to see if the most dependent portion of the pouch will reach past the pubis after it is created extracorporeally, although if the pouch is traversing a thick abdominal pannus, this method of estimation may not be entirely accurate. Various approaches to gaining pouch length are outlined in Table 3.

**Table 3:** Pouch-lengthening techniques

Location	Lengthening technique
Pouch	Creation of S-pouch
Mesentery	Ligate arcade vessel
	Ligate ileocolic vessels
	Ligate SMA (if ileocolic preserved)
	Mobilize SMA to head of pancreas
	Fenestration
Rectal cuff	High transection
Last option	Suture IPAA deep in pelvis

If there is still concern about tension using this approach, there are numerous lengthening procedures which can be employed to improve length. Generally, it is recommended to take the ileocolic vessel at its base during pouch construction. The arcade collaterals are rich, and there is little risk of perfusion. The superior mesenteric artery (SMA) can also be mobilized further by carrying its dissection up to where it passes the head of the pancreas. Particularly in 3-stage pouches, there are often adhesions at this level which can be mobilized to add length.

Once the most dependent portion of the pouch is identified and the pouch is measured out, one can transilluminate the vessels and identify one that may be sacrificed in increase length. This should be a second-order arcade vessel and not one close to the bowel wall itself. If there is concern about sacrificing the vessel, one can apply a bulldog clamp to the vessel and test perfusion, either by waiting and allowing the bowel to demarcate or through ICG fluoroscopy, which is a much faster technique.

If the pouch has not been created yet and there is still significant concern about reach, this is the point at which one can consider employing an S-pouch to help increase length. It is critically important to keep the outflow valve short, at between 1 and 1.5 cm, or there is a high risk of outflow obstruction and incomplete emptying of the pouch. If the pouch has been created and will not reach the anus, the mesentery can be fenestrated to allow for lengthening [64]. It is important to remember that both sides of the mesentery will need to be fenestrated or this likely will not help. Usually, multiple fenestrations are necessary as well to obtain extra length. If the ileocolic vessels have been preserved, the SMA can be ligated distal to the takeoff of these vessels which can preserve the arcade structure while allowing for increased length of the mesentery [65]. Once all of these techniques have been tried and your pouch will not reach the last option to bring the pouch as deep into the pelvis as is possible, tack it into place and complete the procedure with a loop ileostomy in place. Oftentimes the mesentery will stretch over a period of 3–6 months at which time one can come back and gain the extra few centimeters necessary to complete the anastomosis. 

Anastomotic leak is one of the most feared complications of this procedure. We have already discussed staging of pouches to help minimize the risk of pelvic sepsis. While this will not prevent a leak, it will minimize its infectious complications. Often these leaks are identified on postoperative gastrografin enema and, when identified in a diverted asymptomatic patient, will generally heal with time. If a leak is identified and the patient is symptomatic and undiverted, it is generally wise to divert and achieve drainage, either surgically or via a radiologically guided catheter. There is also limited data on the usage of endosponge to treat pouch leaks through an endoscopically placed sponge that is slowly exchanged to downsize. However, this technique can be resource intense and is not widely available [66]. Often the collections resulting from a leak can be drained through the anastomotic defect using a Malecot catheter. This will help minimize the risk of developing a fistula, and the catheter can be serially downsized to allow the collection to seal down around it. This is generally only successful in the diverted patient.

If the patient is diverted and the anastomotic defect is not closing, then other techniques may need to be employed. If the patient is able to develop a stable, epithelialized sinus, then ileostomy closure may be advisable. It is important to determine if there is an overlapping flap of tissue in this setting, however, as this can act as a valve which can impair function. In this setting, endoscopic procedures have been employed to marsupialize this flap and create a stable sinus [67]. Another option is a pouch advancement flap where the cavity is debrided and the pouch is brought down to the level of the anastomosis and sutured into place. If all of these techniques fail and the anastomosis cannot be salvaged, then pouch revision can be offered to well-selected individuals.

There may also be times where an anastomotic defect manifests as a fistula, either a pouch anal fistula or sometimes, in women, a pouch vaginal fistula. When a patient develops a pelvic fistula after a pouch, it is important to first rule out CD. If the patient is over a year out from their surgery, this is unlikely to be a surgical complication; similarly, if the patient is recently out of surgery, this is more likely to be iatrogenic in nature. It is important to start with controlling the infectious complications. This may require an incision and drainage and often may also require a seton to be placed. If the patient is particularly symptomatic or a seton alone is not controlling the infectious complications, then diversion is warranted.

To truly rule out CD, a pouchoscopy is advised. It is important to scope the inflow of the pouch and biopsy any abnormalities. A biopsy where pouchitis is seen is nonspecific, and often the visual findings are more helpful than the pathologic ones. Identifying the internal orifice is important as well as surgical complications will generally be at the staple or suture lines, whereas CD can occur anywhere, and will often come from the retained rectal cuff. If one does feel that the fistula was caused by CD, it is advisable to begin with medical management and obtain maximal medical control prior to considering repair. If the fistula is more likely surgical, some of the previously mentioned methods can be attempted to control the fistula.

Up to 40% of patients will develop pouchitis in the first year after pouch formation with up to an 80% lifetime risk [68]. Pouchitis is characterized by inflammation of the pouch which can lead to frequency, urgency, bloody bowel movements, abdominal pain, or cramping. Generally these patients should be considered for pouchoscopy, and causes of pouchitis, including NSAIDs, infection, radiation, and ischemia, should be ruled out. Patients with PSC also have a higher incidence of pouchitis and should be counseled on this prior to surgery. When the diagnosis of pouchitis is made, it is generally treated with a 2-week course of antibiotics, and symptoms will resolve in the majority of patients. Up to 20% can go on to develop chronic antibiotic-dependent pouchitis (CADP) [69], defined as 4 or more episodes per year responding to antibiotics. A small subset of these will develop chronic antibiotic refractory pouchitis (CARP) which does not respond to antibiotics, and may ultimately require biologics or diversion. There is some evidence to support probiotics, in particular VSL#3, for prevention of pouchitis [70]; however, the data is limited and the company making VSL#3 has been legally forced to change its formulation, and thus, there is some confusion about what the original proprietary formulation included [71].

Cuffitis is caused by recurrent UC in retained columnar mucosa, usually between the dentate line and a stapled anastomosis, and the risk of cuffitis increases with the length of the rectal cuff. Symptoms can appear similar to pouchitis, but these patients may also develop significant tenesmus. Cuffitis is generally well treated with 5-ASA derivative suppositories, but not all patients may respond to these therapies, in which case CD should be ruled out. Surgery may be a last option for patients with refractory cuffitis, where a completion mucosectomy and pouch advancement is performed.

Revisionary surgery after creation of a CI is extremely common and can occur in up to 72% of patients [72]. The most common need for reoperation in this population is valve slippage, which generally presents as either a loss of continence or an inability to cannulate the pouch. While it is possible to salvage a CI after valve slippage, this procedure is often quite complex, involving removal of the valve, reversal of the pouch, and conversion of the pouch inflow to a new outflow valve. This should only be undertaken by those who have significant experience with these procedures as once a valve has been repaired, it is likely to slip again. The Cleveland Clinic noted that patients with valve slippage underwent a median of 2.9 procedures at an average interval of 14 months, although they were able to salvage 77% of these pouches at 20 years [72].

## Conclusion

Surgery for UC includes total proctocolectomy with or without ileal pouch reconstruction. The tenets of surgery in this setting are first controlling the disease, and once a patient is in a suitable risk category, only then should reconstruction be considered. Regardless of one’s preferred minimally invasive technique, outcomes from minimally invasive surgery are good, and this should be the preferred approach at this point in time. Having a good familiarity with the potential complications of pouch creation is critical in managing these patients in the perioperative period as well as for the rest of their lives. If a patient is not a pelvic pouch candidate, a CI may be considered; however, these procedures are complex and carry a high risk of reoperation. There is still no consensus on appendectomy in the setting of UC, but there will be more data on this with time.
